# Diosmetin Restores Endoplasmic Reticulum Homeostasis to Ameliorate Podocyte Injury in Diabetic Kidney Disease by Targeting DNAJC3

**DOI:** 10.1155/jdr/5130183

**Published:** 2026-09-16

**Authors:** Tianyu Zhang, Na Zhao, Tongjin Liu, Yamei Gao, Zhiyue Di, Yue Gao, Yanan Ji, Shuquan Lv, Qingying Xie, Jian Ma, Huantian Cui

**Affiliations:** ^1^ First School of Clinical Medicine, Heilongjiang University of Chinese Medicine, Harbin, China, hljucm.edu.cn; ^2^ Department of Endocrinology, First Affiliated Hospital of Heilongjiang University of Chinese Medicine, Harbin, China, hljucm.edu.cn; ^3^ Graduate School, Cangzhou Hospital of Integrated Traditional Chinese and Western Medicine of Hebei Province, Cangzhou, China; ^4^ Department of General Internal Medicine III, Harbin Hospital of Traditional Chinese Medicine, Harbin, China; ^5^ Department of Respiratory Medicine, First Affiliated Hospital of Heilongjiang University of Chinese Medicine, Harbin, China, hljucm.edu.cn; ^6^ Department of Endocrinology, Cangzhou Hospital of Integrated Traditional Chinese and Western Medicine of Hebei Province, Cangzhou, China; ^7^ First School of Clinical Medicine, Yunnan University of Chinese Medicine, Kunming, China, ynutcm.edu.cn

**Keywords:** diabetic kidney disease, diosmetin, DNAJC3, endoplasmic reticulum stress, podocyte

## Abstract

**Background:**

Diabetic kidney disease (DKD) causes high mortality and imposes a substantial healthcare burden, representing a major global public health challenge. The natural flavonoid diosmetin (DIO) shows promising therapeutic effects in DKD; however, its molecular mechanisms remain unclear. This study is aimed at clarifying the specific mechanisms and molecular targets through which DIO exerts its effects in DKD.

**Methods:**

We established an in vitro podocyte injury model using high glucose (HG) and palmitic acid (PA) to evaluate the effects of DIO on podocyte viability. We performed transcriptomic analysis to identify potential mechanisms underlying the protective effects of DIO on podocytes, and we validated key pathway‐related proteins by Western blot. We applied molecular docking, cellular thermal shift assay (CETSA), and drug affinity responsive target stability (DARTS) to identify potential target proteins, and we further validated these targets using shRNA technology. Finally, we established a DKD mouse model by combining a high‐fat/high‐sugar diet with streptozotocin (STZ) administration to assess the therapeutic effects of DIO and its capacity to alleviate endoplasmic reticulum stress (ERS).

**Results:**

DIO significantly attenuated HG/PA–induced podocyte injury and restored podocyte viability. Gene set enrichment analysis (GSEA) showed that the MISFOLDED_PROTEIN_BINDING pathway, which is closely associated with ERS regulation, was upregulated after DIO treatment. At the molecular level, DIO increased the expression of DNAJC3, a key regulator of endoplasmic reticulum homeostasis within this pathway, and reduced the levels of ERS‐related proteins, including GRP78, P‐eIF2*α*/eIF2*α*, ATF4, and CHOP. Molecular interaction analyses demonstrated that DIO has a strong binding affinity for DNAJC3, indicating a direct targeting effect. Knockdown of DNAJC3 markedly weakened the protective effects of DIO on podocytes. In vivo, DIO treatment significantly improved hyperglycemia, proteinuria, renal pathological injury, and oxidative stress in DKD mice. Notably, DIO also enhanced DNAJC3 expression and suppressed ERS in DKD mice.

**Conclusions:**

This study clarifies the molecular basis of DIO‐mediated protection in DKD, particularly in podocytes, by identifying DNAJC3 as a potential target that DIO inhibits ERS, restores ER homeostasis, and alleviates podocyte injury.

## 1. Introduction

Diabetic kidney disease (DKD) is a chronic complication of diabetes mellitus (DM), manifested by persistent albuminuria and/or a decline in glomerular filtration rate. According to the latest statistics from the IDF Diabetes Atlas (11th edition), the global number of adults with diabetes reached 589 million in 2024 and might be as high as 853 million by 2050 [1]. Among these individuals, approximately 20%–50% will develop DKD, as the leading cause of end‐stage renal disease worldwide and remains a major contributor to mortality [2, 3]. To date, DKD has markedly reduced patients′ quality of life and imposed a substantial economic burden on healthcare systems.

The pathogenesis of DKD involves the coordinated dysregulation of multiple signaling pathways, including inflammation, oxidative stress, apoptosis, and endoplasmic reticulum stress (ERS) [4]. These interconnected mechanisms drive disease progression and increase the complexity of effective intervention. Current clinical management of DKD primarily focuses on symptom control, including glycemic regulation, blood pressure reduction, and mitigation of proteinuria [5]. In recent years, agents such as losartan and finerenone have shown clear benefits in preserving renal function and reducing proteinuria [6]. However, their clinical utility may be limited by high treatment costs and challenges in long‐term patient adherence. Therefore, identifying novel and effective therapeutic strategies for DKD remains an urgent priority and a major challenge in contemporary biomedical research.

Over the past few decades, researchers have increasingly recognized the critical role of the endoplasmic reticulum (ER) in the pathogenesis of DKD. The ER serves as a central organelle for protein synthesis, protein folding, and lipid metabolism, and its functional integrity is essential for cell survival [7]. Under chronic exposure to hyperglycemia, oxidative stress, and inflammatory stimuli, unfolded or misfolded proteins accumulate within the ER lumen [8]. This accumulation disrupts ER function and further amplifies oxidative stress, inflammatory responses, and apoptosis [9]. The resulting disruption of ER homeostasis, caused by failure of adaptive ER defense mechanisms, is defined as ERS. Clinical studies have shown that ERS in the kidney progressively intensifies as DKD advances [10]. Among renal intrinsic cells, glomerular podocytes play a key role in maintaining the structural and functional integrity of the glomerular filtration barrier, and their injury represents a central event in DKD progression [11]. A reduction in podocyte density is currently considered the strongest predictor of DKD progression and shows a direct correlation with the severity of proteinuria [12]. Notably, podocytes contain a highly developed ER to support their substantial protein‐folding demands, which makes them particularly vulnerable to ERS [12]. Accumulating evidence from both in vitro and in vivo studies demonstrates that inhibition of ERS can effectively repair podocyte injury and improve podocyte function [13–15]. Therefore, preserving ER homeostasis in podocytes and preventing excessive ERS activation represent promising and rational therapeutic strategies for DKD.

Traditional Chinese medicine (TCM)–derived natural products, characterized by multitarget regulation and relatively low toxicity, have shown considerable potential in the prevention and treatment of DKD [16, 17]. Several such compounds, including astragaloside IV [14], berberine [18], emodin [19], triptolide [20], Hydroxysafflor yellow A [21], and phillygenin [22], have demonstrated protective effects against podocyte injury in DKD. Given that oxidative stress and inflammatory responses are key drivers of podocyte damage, the natural flavonoid DIO, which possesses both antioxidant and anti‐inflammatory properties, has attracted increasing attention. Previous studies have shown that DIO exerts antioxidant and anti‐inflammatory effects in various animal disease models [23–25], displays favorable pharmacokinetic characteristics [26], and provides therapeutic benefits in DKD [27]. However, its mechanisms of action in DKD, particularly its protective effects on podocytes and its direct molecular targets, remain unclear. With the advancement of omics technologies, correlation analysis integrating DKD key genes with clinical phenotypes has emerged as an effective approach for screening novel biomarkers and potential therapeutic targets [28].

In this study, we first established an in vitro podocyte injury model induced by HG/PA to assess the protective effects of DIO. We then performed transcriptomic sequencing to analyze changes in gene expression in the HG/PA–induced podocyte injury model following DIO treatment, with the aim of identifying potential signaling pathways and target molecules. Next, we applied molecular docking, CETSA, and DARTS to evaluate the interactions between DIO and candidate target proteins, and we further validated these targets through gene knockdown experiments in vitro. In addition, we established a DKD mouse model using a high‐sugar/high‐fat diet combined with STZ injection to examine the therapeutic effects of DIO in vivo, as well as its protective effects on podocytes and its regulatory role in ERS. This study preliminarily clarifies a novel mechanism by which DIO protects against DKD by alleviating ERS in podocytes, thereby providing a foundation for expanding the pharmacological applications of DIO.

## 2. Materials and Methods

### 2.1. Cells and Reagents

**Figure 1:**
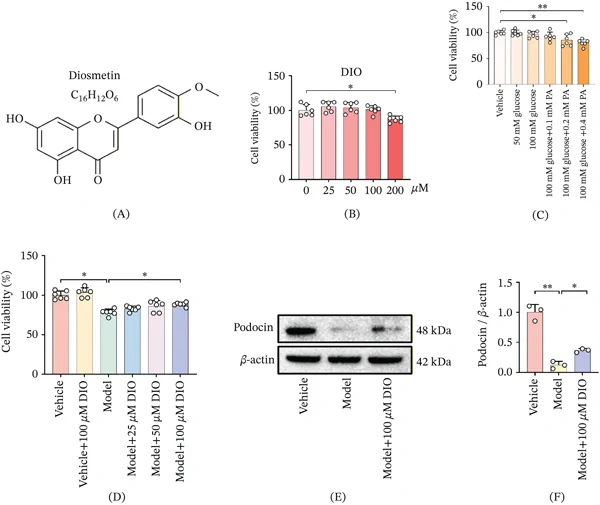
DIO ameliorates podocyte injury induced by combined HG/PA treatment. (A) Chemical structure of DIO. (B) Effects of DIO on MPC5 cell viability. (C) Comparison of effects on MPC5 cell viability between combined glucose/PA and HG‐alone interventions. (D–F) Cell viability and Podocin expression in each group. Data are presented as means ± SD. *n* = 6 per group for (B–D), *n* = 3 per group for (E,F). ∗*p* < 0.05, ∗∗*p* < 0.01∗∗*p* < 0.01.

DIO (HY‐N0125, C_16_H_12_O_6_, purity > 99*%*) was used in this study, and its molecular structure is shown in Figure 1A. Detailed information on other experimental drugs and reagents is provided in the Supporting Information.

### 2.2. Cell Culture and Viability Assay

Mouse podocyte MPC5 cells (Catalog Number nobcell0552, source: Zhejiang Noble Biological Products Co., Ltd.) were cultured in RPMI 1640 medium supplemented with 10% fetal bovine serum (FBS) and 10‐U/mL interferon‐gamma (IFN‐*γ*) at 33°C in a 5% CO_2_ incubator to maintain proliferation. To induce differentiation, we transferred the cells to IFN‐*γ*‐free medium and cultured them at 37°C for 10 days.

To determine the appropriate concentrations of DIO and establish modeling conditions, we seeded MPC5 cells in the logarithmic growth phase into 96‐well plates at a density of 1 × 10^4^ cells per well. We then treated the cells with different concentrations of DIO (0, 25, 50, 100, 200 *μ*M) for 24 h. To determine the modeling dosage for the MPC5 injury model, we treated the cells with glucose (5.5, 50, and 100 mM) and PA (0, 0.1, 0.2, and 0.4 mM) for 24 h. We assessed cell viability using the MTT assay by measuring OD_490_ [29].

### 2.3. Establishment of the Cell Model

We established a podocyte injury model using combined HG and PA treatment [30, 31]. Differentiated MPC5 cells were exposed to 100‐mM glucose and 0.4 mM PA (HG/PA) for 24 h. We divided the cells into six groups: control group (treated with 5.5‐mM glucose, vehicle), drug‐alone group (treated with 5.5‐mM glucose and 100 *μ*M DIO, vehicle + DIO), model group (treated with HG/PA, Model), and DIO treatment group (treated with HG/PA and 100 *μ*M DIO, Model + 100 *μ*M DIO). DIO was administered simultaneously with 5.5‐mM glucose or HG/PA treatment.

### 2.4. Transcriptomics

We selected cells from the vehicle, model, and model + 100 *μ*M DIO groups for transcriptomic analysis, *n* = 3 biological replicates per group. We extracted total RNA using the Trizol method. Novogene Co., Ltd. performed RNA library construction and sequencing according to standard protocols [32]. We identified differentially expressed genes (DEGs) using the criteria |Log(*f* *o* *l* *d* *c* *h* *a* *n* *g* *e*)| > 1 and *p* value < 0.05. We then performed Gene Ontology (GO) analysis and GSEA on the identified DEGs. The methodological details related to the transcriptomic analysis are provided in Supporting Information.

### 2.5. Molecular Docking

We obtained the molecular structure of DIO from the PubChem database and the three‐dimensional structure of DNAJC3 from the UniProt database. We removed water molecules and predicted the active binding pocket using AutoDock Tools. We then performed molecular docking using AutoDock Vina.

### 2.6. CETSA and DARTS

After HG/PA induction, we collected MPC5 cells, resuspended them and subjected them to repeated freeze–thaw cycles. We performed CETSA and DARTS to evaluate the binding stability between DIO and DNAJC3, following previously established protocols [33].

### 2.7. Silencing of DNAJC3 in MPC5 Cells

We seeded differentiated MPC5 cells into 24‐well plates at a density of 4 × 10^4^ cells per well. We incubated Lipo3000 transfection reagent with shDNAJC3 or shNC in medium for 6 h, then replaced it with fresh medium without transfection reagent or shRNA and continued incubation. The sequence of shDNAJC3‐1 was: SS ‐ AGAAAGUGCUCAAAUCUAACC, AS ‐ UUAGAUUUGAGCACUUUCUUG; and the sequence of shDNAJC3‐2 was: SS ‐ CGAGCAGAAUGCUUCAUAAAG, AS ‐ UUAUGAAGCAUUCUGCUCGCA.

### 2.8. Animal Modeling and Grouping

Male C57BL/6 mice of specific pathogen‐free (SPF) grade, weighing 18–22 g, were obtained from SiPeiFu (Beijing) Biotechnology Co., Ltd. (Experimental Animal Production License Number SCXK(Jing)2024‐0001). All animal experimental protocols were approved by the Medical Ethics Committee of Cangzhou Hospital of Integrated Traditional Chinese and Western Medicine of Hebei Province (Approval Number CZX2025‐DW005‐01), and all procedures complied with national guidelines for the care and use of laboratory animals. Before the experiments, the mice were allowed to acclimatize for 1 week.

We randomly assigned a total of 50 mice into 5 groups (*n* = 10 per group): normal group (Control), DKD model group (DKD), irbesartan (IRB) treatment group, low‐dose diosmetin group (L‐DIO), and high‐dose diosmetin group (H‐DIO). Except for Control, we induced DKD in all groups following established protocols [34]. Briefly, we fed the mice a high‐sugar/high‐fat diet for 8 weeks. At the end of Week 8, we administered STZ intraperitoneally at a dose of 30 mg/kg once daily for 3 consecutive days. In parallel, the Control group received an equivalent volume of sodium citrate buffer via intraperitoneal injection. Three days after STZ administration, we measured fasting blood glucose (FBG) levels. Mice with FBG > 11.1 mmol/L were considered to have successfully developed DM. They were then continuously fed a high‐sugar and high‐fat diet. DKD mouse models were considered successfully established when FBG > 11.1 mmol/L and ACR was significantly elevated [35]. After successful DKD modeling, we treated the IRB group with IRB [36] (30 mg/kg/day) by oral gavage. We administered diosmetin to the L‐DIO and H‐DIO groups at doses of 12.5 and 50 mg/kg, respectively, by daily gavage. Both IRB and DIO were dissolved in 0.5% CMC‐Na. The Control and DKD groups received an equal volume of 0.5% CMC‐Na by gavage. All treatments were continued for 8 weeks.

During treatment, we measured blood glucose levels and body weight weekly. At the end of the 8‐week treatment period, we fasted the mice. We then anesthetized the mice and collected blood samples via the medial canthus. After sample collection, we euthanized the mice and harvested both kidneys. We fixed the right kidney in 4% paraformaldehyde, whereas we stored the left kidney at −80°C for subsequent analyses.

### 2.9. Biochemical Parameter Detection

We measured serum creatinine (Cr) and blood urea nitrogen (BUN) levels in each group. We also collected urine samples to quantify the ratio of urinary albumin to creatinine (ACR; *μ*g/mg). ACR was calculated according to the following formula: ACR = urinary albumin (*μ*g/dL)/urinary Cr (*μ*g/dL). We also assessed oxidative stress markers in renal tissue, including superoxide dismutase (SOD), malondialdehyde (MDA), and glutathione peroxidase (GSH‐Px) [37]. All assays were performed strictly according to the manufacturers′ instructions.

### 2.10. Pathological Staining

We dehydrated, embedded, and sectioned renal tissues into 4‐*μ*m slices. We then performed H&E and periodic acid–Schiff (PAS) staining [38]. Blinding was used in the histopathological analysis. Briefly, two independent pathologists evaluated the histological sections using a pre‐established standardized scoring system. Samples were identified solely by experimental numbers, and both pathologists remained blinded to the group assignments. We conducted quantitative analysis using ImageJ software.

### 2.11. RT‐qPCR

We extracted total RNA from renal tissues of each group using a commercial kit and reverse‐transcribed it into cDNA. We measured mRNA expression levels of target genes by qPCR. We calculated relative mRNA expression using the 2^−*Δ* *Δ*CT^ method [39]. The primer sequences used in this study are listed in Table S1.

### 2.12. Western Blot

We extracted total protein from renal tissues or cultured cells and separated the proteins by electrophoresis, followed by wet transfer onto polyvinylidene fluoride (PVDF) membranes. We blocked the membranes for 2 h and then incubated them with primary antibodies against Podocin, DNAJC3, GRP78, P‐eIF2*α*, eIF2*α*, ATF4, and CHOP at 4°C for 12 h. After washing, we incubated the membranes with appropriate secondary antibodies at 37°C for 2 h. We then washed the membranes again and added the chemiluminescent developing solution. We visualized protein bands using an automated gel imaging system and quantified band intensities using ImageJ software. We determined total protein concentrations using the BCA assay, with *β*‐actin as the internal control.

### 2.13. Statistical Analysis

We performed statistical analysis using the SPSS Pro online data analysis platform. Data are presented as mean ± standard deviation. All datasets passed the Shapiro–Wilk test for normality. We analyzed differences among groups using one‐way analysis of variance (ANOVA), followed by the LSD‐t test for pairwise comparisons. *p* < 0.05 was considered statistically significant.

## 3. Results

### 3.1. DIO Ameliorates HG/PA–Induced Podocyte Injury

To determine nontoxic concentrations of DIO for MPC5 cells, we used the MTT assay to assess cell viability after treatment with different concentrations of DIO. DIO at concentrations below 100 *μ*M showed no cytotoxic effects (Figure 1B). In parallel, we treated MPC5 cells with various concentrations of glucose or PA to identify suitable conditions for model induction. Below 100‐mM glucose alone did not significantly affect podocyte viability compared with the vehicle group. However, when combined with PA, cell viability decreased in a dose‐dependent manner. Specifically, 100 − mM glucose + 0.4 − mM PA caused a robust decrease in podocyte viability (Figure 1C). Therefore, we used the combined 100 − mM glucose + 0.4 − mM PA (HG/PA) treatment to establish the podocyte injury model and treated cells with 100 *μ*M DIO. The results showed that DIO improved podocyte viability and Podocin expression under HG/PA conditions (Figure 1D−F), indicating its protective effect in vitro.

### 3.2. DIO Inhibits ERS in Podocytes

**Figure 2:**
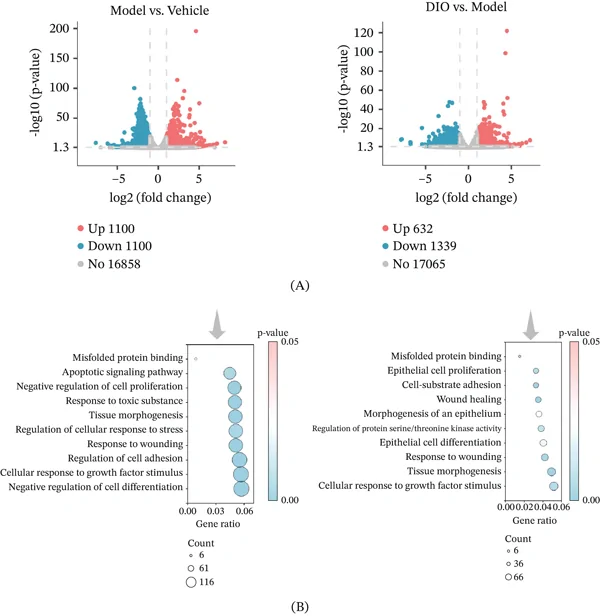
Effects of DIO on the transcriptome of podocytes induced by combined HG/PA treatment. (A) Volcano plots of gene expression in the model versus vehicle and DIO versus model comparisons. (B) GO enrichment analysis of DEGs.

We next performed transcriptomic analysis on MPC5 cells from the vehicle, model, and model + DIO groups. We visualized DEGs from the comparisons of model versus vehicle and DIO versus model using volcano plots (Figure 2A). GO enrichment analysis, based on a *p* value < 0.05, showed significant enrichment of injury repair and cell morphology‐related pathways, including misfolded protein binding, tissue morphogenesis, response to wounding, and cellular response to growth factor stimulus (Figure 2B).

**Figure 3:**
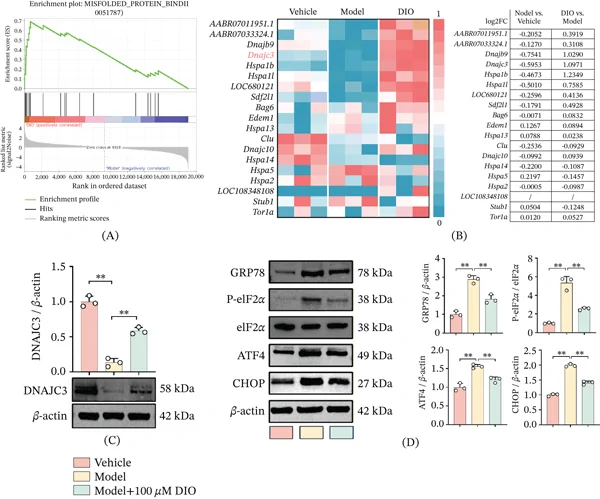
DIO inhibits ERS in podocytes. (A) GSEA analysis revealing enrichment in the MISFOLDED_PROTEIN_BINDING pathway. (B) Heatmap of gene expression related to the MISFOLDED_PROTEIN_BINDING pathway. (C) Western blot analysis showing that DIO intervention upregulates DNAJC3 protein levels in podocytes induced by HG/PA. (D) Western blot analysis showing that DIO intervention reduces protein levels of GRP78, P‐eIF2*α*/eIF2*α*, ATF4, and CHOP in podocytes induced by HG/PA. ∗∗*p* < 0.01.

Further GSEA showed that, compared with the model group, treatment with DIO significantly upregulated the MISFOLDED_PROTEIN_BINDING pathway, which is closely associated with ERS. To further examine this pathway, we analyzed the expression of related genes and generated a heatmap for the DIO versus model comparison (Figure 3A and Table S2). The heatmap showed that DIO increased the expression of several genes, including *Sdf2l1, Hspa1l, Dnajb9, AABR07033324.1, Dnajc3, AABR07011951.1, LOC680121*, and *Hspa1b*. Among these, *Dnajc3* was identified as the second most highly upregulated transcript following DIO treatment (Figure 3B). Notably, DNAJC3 has been reported to inhibit ERS in DKD models, and its upregulation suppresses the expression of ERS‐related proteins, including GRP78, P‐eIF2*α*/eIF2*α*, ATF4, and CHOP [40–42]. We then performed Western blot analysis to measure the protein levels of DNAJC3, GRP78, P‐eIF2*α*/eIF2*α*, ATF4, and CHOP. The results showed that DIO increased DNAJC3 protein expression in HG/PA–induced podocytes and reduced the expression of GRP78, P‐eIF2*α*/eIF2*α*, ATF4, CHOP proteins (Figure 3C,D), indicating effective inhibition of ERS.

### 3.3. DIO Exerts Protective Effects on HG/PA–Induced Podocytes by Targeting DNAJC3

**Figure 4:**
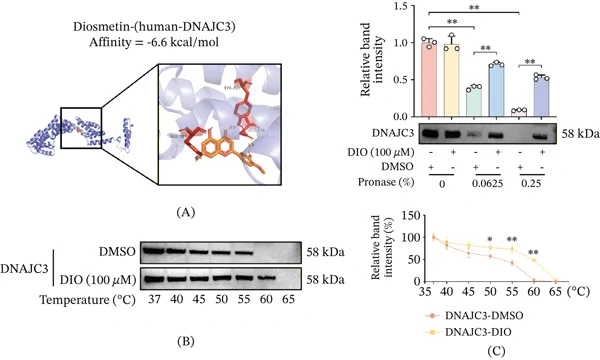
DIO targets DNAJC3. (A) Molecular docking predicting binding affinity between DIO and DNAJC3. (B) CETSA demonstrating that DIO enhances thermal stability of DNAJC3. (C) DARTS showing that DIO enhances resistance of DNAJC3 to proteolysis. ∗*p* < 0.05, ∗∗*p* < 0.01.

Based on the regulatory role of DNAJC3 identified in the podocyte injury model through transcriptomic analysis, we hypothesized that DNAJC3 may act as a key target of DIO in DKD. We first performed molecular docking to evaluate the interaction between DIO and DNAJC3. The calculated binding energy was −6.6 kcal/mol (Figure 4A), indicating a strong binding affinity. We further validated this interaction using CETSA and DARTS assays. Compared with the DMSO‐treated group, DIO treatment increased the thermal stability and protease resistance of DNAJC3 (Figure 4B,C), confirming stable binding between DIO and DNAJC3.

**Figure 5:**
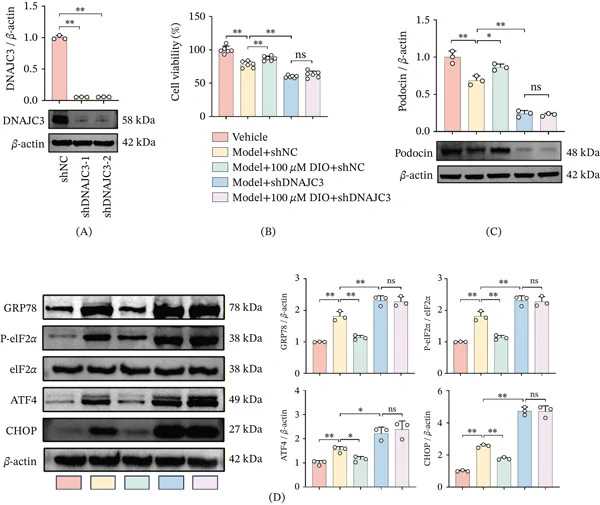
Silencing DNAJC3 attenuates protective effects of DIO on podocytes induced by HG/PA. (A) Silencing efficiency of shDNAJC3. After silencing DNAJC3, the enhancing effect of DIO on the (B) viability and (C) Podocin expression of injured podocytes, and (D) its inhibitory effects on the protein expression of GRP78, P‐eIF2*α*/eIF2*α*, ATF4, and CHOP are significantly weakened or abolished. ns: no significant, ∗*p* < 0.05, ∗∗*p* < 0.01.

We further silenced *Dnajc3* gene in MPC5 cells and selected sh*DNAJC3*‐1 for subsequent experiments based on its higher silencing efficiency (Figure 5A). After *DNAJC3* knockdown, we induced podocyte injury using HG/PA and treated the cells with 100 *μ*M DIO. The results showed that *DNAJC3* silencing markedly weakened the ability of DIO to restore MPC5 cell viability and promote Podocin expression (Figure 5B,C). In addition, *DNAJC3* knockdown almost completely abolished the inhibitory effects of DIO on ERS‐related proteins, including GRP78, P‐eIF2*α*/eIF2*α*, ATF4, and CHOP (Figure 5D). These findings confirm that DNAJC3 mediates the protective effects of DIO against DKD.

### 3.4. DIO Ameliorates DKD

**Figure 6:**
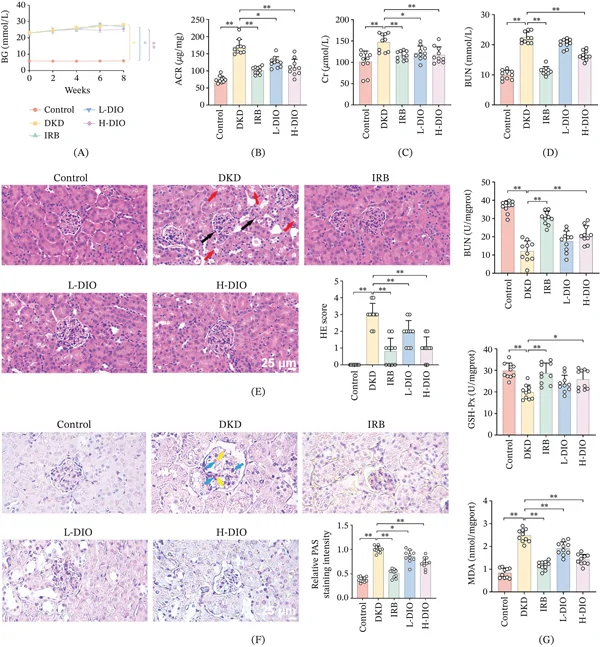
Therapeutic effects of DIO on DKD mice. (A) DIO improves FBG levels, (B–D) reduces ACR, Cr, and BUN levels, (E,F) alleviates renal histopathological changes in DKD mice, and (G) decreases oxidative stress levels. Red arrow indicates tubular vacuolar degeneration, black arrow indicates interstitial inflammatory infiltration, yellow arrow indicates thickened glomerular basement membrane, and blue arrow indicates expanded mesangial matrix. ∗*p* < 0.05, ∗∗*p* < 0.01.

We further validated the therapeutic effects of DIO in vivo using a DKD mouse model induced by a high‐sugar/high‐fat diet combined with STZ. During the treatment period, we monitored FBG levels weekly. At the end of the experiment, FBG levels in the DKD group were significantly higher than those in the control group, whereas they were reduced in the H‐DIO group (Figure 6A). Renal function analysis showed that levels of ACR, Cr, and BUN were significantly elevated in the DKD group. Treatment with DIO reduced these indicators to varying degrees in both the L‐DIO and H‐DIO groups (Figure 6B–D). Histopathological examination further supported these findings. H&E staining revealed severe renal damage in DKD mice, including glomerular hypertrophy and tubular vacuolar degeneration. PAS staining showed thickening of the glomerular basement membrane and increased deposition. In contrast, treatment with IRB and DIO markedly alleviated these pathological changes. DIO also reduced renal injury scores and glomerulosclerosis scores (Figure 6E,F). Analysis of oxidative stress markers showed that DKD mice exhibited significantly decreased levels of SOD and GSH‐Px, along with increased levels of MDA in renal tissues (Figure 6G). DIO treatment effectively reversed these changes, indicating an improvement in oxidative stress status.

### 3.5. DIO Ameliorates ERS In Vivo

**Figure 7:**
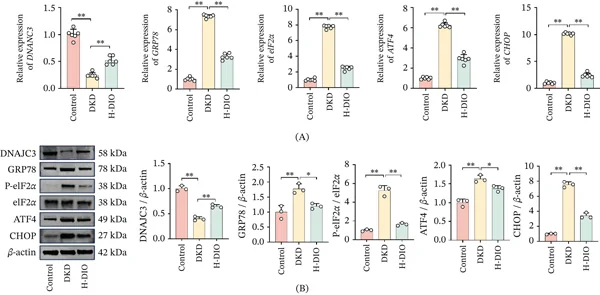
DIO inhibits ERS in DKD mice. (A) RT‐qPCR analyses showing that DIO upregulates DNAJC3 while downregulating GRP78, eIF2*α*, ATF4, and CHOP gene levels in DKD mice renal tissues. (B) Western blot showing that DIO increases DNAJC3 expression while reducing GRP78, P‐eIF2*α*/eIF2*α*, ATF4, and CHOP protein expression in DKD mice renal tissues. ∗*p* < 0.05, ∗∗*p* < 0.01.

Finally, we assessed the effects of DIO on ERS in renal tissues from DKD mice. RT‐qPCR analysis showed that DIO significantly increased DNAJC3 mRNA expression and decreased the mRNA levels of ERS‐related genes, including GRP78, eIF2*α*, ATF4, and CHOP, in DKD renal tissues (Figure 7A). Western blot analysis further confirmed these changes at the protein level (Figure 7B). These results indicate that DIO exerts its anti‐DKD effects by targeting DNAJC3 and suppressing ERS‐related signaling, including GRP78, P‐eIF2*α*/eIF2*α*, ATF4, and CHOP.

## 4. Discussion

DKD is one of the most severe microvascular complications of diabetes, and persistent hyperglycemia is a central driver of its onset and progression. Podocytes are highly differentiated terminal cells that play a critical role in maintaining the integrity of the glomerular filtration barrier. Under prolonged metabolic stress, podocytes experience disruption of protein homeostasis, which leads to sustained ERS and promotes apoptosis and detachment [43]. Therefore, restoring protein homeostasis in podocytes is essential for slowing DKD progression. In this study, we established an in vitro podocyte injury model using combined HG/PA stimulation, a widely accepted approach that mimics the DKD microenvironment [44]. Our results showed that DIO treatment significantly improved the viability of injured podocytes, providing initial evidence for its therapeutic potential in DKD.

To further elucidate the molecular mechanisms underlying the protective effects of DIO on podocytes, we performed transcriptomic sequencing. GO enrichment analysis identified several key biological processes related to injury repair that were regulated by DIO. GSEA further revealed that the MISFOLDED_PROTEIN_BINDING pathway represents a critical functional component of DIO‐mediated protection. Aberrant activation of this pathway reflects disruption of cellular protein homeostasis and indicates the presence of ERS [45]. Notably, DIO treatment significantly upregulated the core gene *DNAJC3* within this pathway. *DNAJC3* (DNAJ homolog subfamily C member 3) is an ER‐resident cochaperone of BIP (also known as HSPA5 or GRP78) and plays an essential role in the negative feedback regulation of ERS during the late phase, thereby promoting restoration of cellular homeostasis [46]. GRP78 acts as a central regulator of ERS. Under physiological conditions, GRP78 binds to ER membrane‐associated stress sensors to maintain ER homeostasis [47]. Upon accumulation of misfolded proteins, GRP78 dissociates from ER membrane–associated stress sensors and suppresses global protein translation through phosphorylation of eIF2‐*α* (eukaryotic initiation factor 2 alpha), thereby reducing the protein‐folding burden in the ER [48], while selectively enhancing the translation of the transcription factor ATF4 [49]. When ERS persists, excessive ATF4 accumulation induces the expression of its downstream target CHOP, ultimately activating apoptotic signaling pathways [49].

*DNAJC3* plays a critical role in blocking this signaling cascade, and it is a J‐domain cochaperone that suppresses eIF2*α* phosphorylation and prevents sustained activation of the pro‐apoptotic ATF4/CHOP pathway [50, 51]. Loss‐of‐function mutations of DNAJC3 are closely linked to the onset and progression of diabetes. Clinical evidence shows that individuals carrying DNAJC3 loss‐of‐function mutations develop early‐onset diabetes accompanied by low body weight [40]. Consistently, animal studies have demonstrated that *DNAJC3* knockout mice exhibit hyperglycemia, glucosuria, and increased cellular apoptosis [51]. Under sustained HG/PA stimulation, podocytes undergo prolonged ERS [52], which promotes the degradation of ER luminal proteins, including DNAJC3, via endoplasmic reticulum–associated degradation (ERAD) and the ubiquitin‐proteasome pathway. This accounts for the reduced DNAJC3 levels in the model group. Importantly, DNAJC3 expression is reduced but not abolished; CETSA and DARTS results demonstrated that DIO directly binds the residual DNAJC3, enhancing its thermal stability and resistance to proteolysis. This stabilization allows DNAJC3 to gradually reaccumulate. Because DNAJC3 acts as a catalytic cochaperone rather than a substrate, even partial restoration of its protein level is sufficient to suppress eIF2*α* phosphorylation, thereby alleviating ERS. This in turn reduces ERAD‐mediated degradation pressure, forming a positive feedback loop that further restores DNAJC3 abundance. In our study, DIO treatment reduced eIF2*α* phosphorylation and decreased the expression of ATF4 and CHOP in injured podocytes. Silencing *DNAJC3* in vitro significantly weakened the ERS‐inhibitory and antiapoptotic effects of DIO in podocytes, confirming that DNAJC3 serves as a key target of DIO in regulating ERS. These findings collectively suggest that DIO alleviates ERS‐induced damage and promotes podocyte survival by upregulating DNAJC3. Of note, future research involving DNAJC3 rescue/overexpression or pharmacological modulation of ERS would further strengthen the evidence for the role of DNAJC3 in the protective effects of DIO.

To further validate the in vivo pharmacological effects and underlying mechanisms of DIO, we established a DKD mouse model using a high‐fat diet combined with STZ injection. This model successfully reproduced key clinical and pathological features of DKD, including significant increases in FBG, Cr, BUN, and ACR, along with typical renal pathological changes such as glomerular hypertrophy and basement membrane thickening [53]. DIO treatment markedly reversed these biochemical abnormalities and alleviated renal pathological damage. In addition, DIO significantly improved oxidative stress status in renal tissues. Previous studies have demonstrated that oxidative stress can induce the accumulation of misfolded proteins, thereby triggering ERS [54]. Importantly, DIO upregulated DNAJC3 expression in the kidneys of DKD mice and reduced the levels of ERS‐related markers. However, ERS is not merely a passive response; activated UPR can increase the demand for protein folding, stimulate mitochondrial activity [54, 55], suppress antioxidant gene expression while inducing apoptosis [56], and further exacerbate oxidative stress. Consequently, oxidative stress and ERS form a vicious cycle of mutual promotion and progressive amplification. Taken together, these findings indicate that DIO inhibits excessive ERS and oxidative stress by targeting and enhancing DNAJC3 expression. Through these mechanisms, DIO protects podocytes and delays the progression of DKD in vivo.

In summary, this study demonstrates the therapeutic effects of DIO in DKD and preliminarily elucidates its molecular mechanism in alleviating podocyte ERS through DNAJC3 based on in vitro transcriptomic analysis. Unlike previous studies on anti‐ERS natural compounds, such as astragaloside IV [57], hirudin [58], and terpene glycoside [59], which have largely focused on attenuating ERS‐related inflammatory responses, our findings reveal that DIO directly engages DNAJC3 to restore ER homeostasis rather than relying solely on general antioxidant properties. To our knowledge, this study represents the first identification of DNAJC3 as a pharmacological target of a natural flavonoid for DKD therapy.

However, several limitations remain. First, in addition to *DNAJC3*, several other DEGs related to the misfolded protein‐binding and ER quality control pathways exhibited significant alterations following DIO treatment, such as *Dnajb9, Sdf2l1, Hspa1b*, and *Hspa1l*. Extensive studies have confirmed that these genes are essential components of proteostasis [60–63]. However, whether DIO exerts podocyte‐protective effects by targeting these proteins remains to be further elucidated in future studies. Second, DKD is a multifactorial and progressive disease involving multiple renal compartments, including the glomeruli, renal tubules, interstitium, and microvasculature. Whether DIO produces synergistic protective effects on other intrinsic renal cell types such as glomerular mesangial cells and glomerular endothelial cells [64], as well as whether DIO can regulate inflammation‐associated immune cells such as macrophages [65], remains an important question for further investigation. Third, in addition to the current target validation, performing affinity pull‐down assays would furnish more compelling evidence for direct target engagement. Furthermore, in the present study, DIO was coadministered with HG/PA during in vitro modeling, whereas in vivo DIO treatment was given after DKD establishment. Whether DIO was tested as a preventive or therapeutic intervention can be studied in the future to advance the clinical translation of DIO. Despite these limitations, this study highlights the significant therapeutic potential of targeting the DNAJC3‐mediated ERS pathway in DKD and provides a solid experimental and theoretical foundation for further mechanistic studies and future clinical translation.

## 5. Conclusion

**Figure 8:**
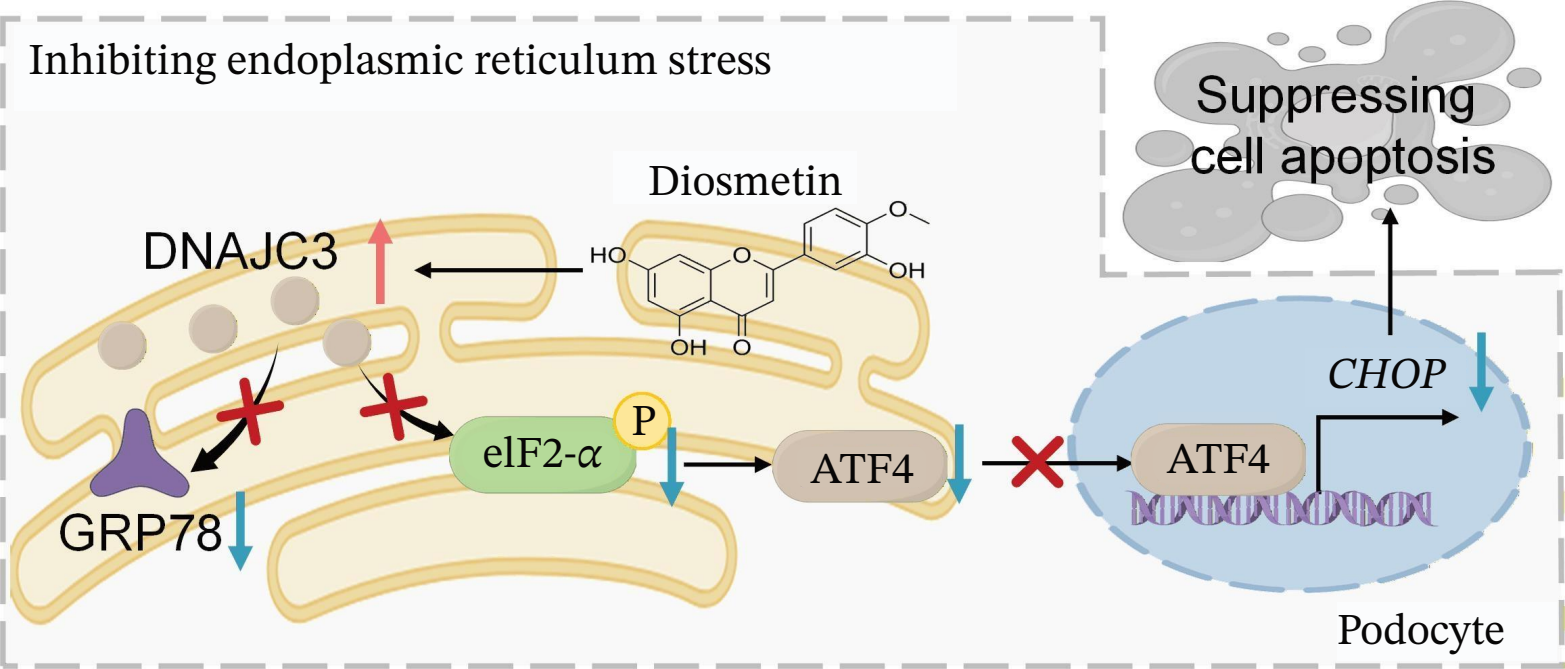
Diosmetin improves podocyte injury in DKD by targeting DNAJC3 to restore endoplasmic reticulum homeostasis.

In conclusion, this study defines the molecular mechanism by which DIO attenuates podocyte injury in DKD by targeting DNAJC3, thereby restoring ER homeostasis. Our findings establish a central role for DNAJC3‐mediated inhibition of ERS in the renoprotective effects of DIO and identify DNAJC3 as a promising therapeutic target for DKD (Figure 8). Future preclinical studies will provide a critical foundation for the clinical translation of this approach.

## Author Contributions

**Tianyu Zhang:** writing – original draft, conceptualization, funding acquisition. **Na Zhao:** writing – original draft, methodology, data curation. **Tongjin Liu:** data curation, formal analysis. **Yamei Gao:** data curation, validation. **Zhiyue Di:** data curation, validation. **Yue Gao:** investigation, data curation. **Yanan Ji:** formal analysis, validation. **Shuquan Lv:** formal analysis. **Qingying Xie:** formal analysis. **Jian Ma:** conceptualization, writing – review & editing. **Huantian Cui:** conceptualization, writing – review & editing, project administration.

## Funding

This study was supported by the Hebei Natural Science Foundation (H2025110036) and Science and Technology Program of Hebei Province (262W7713D).

## Disclosure

All authors have read and approved the final version of the manuscript. Jian Ma and Huantian Cui have full access to all of the data in this study and take complete responsibility for the integrity of the data and the accuracy of the data analysis.

## Ethics Statement

The experimental protocol was approved by the Medical Ethics Committee of Cangzhou Hospital of Integrated Traditional Chinese and Western Medicine of Hebei Province (Approval Number CZX2025‐DW005‐01), and all procedures complied with national guidelines for the care and use of laboratory animals.

## Conflicts of Interest

The authors declare no conflicts of interest.

## Supporting Information

Additional supporting information can be found online in the Supporting Information section.

## Supporting information


**Supporting Information 1** File S1: The details of materials and reagents can be found in the reagents. The details of transcriptomics can be found in the Supporting Information methods. Table S1: Primer sequence used in RT‐qPCR experiment of this study. Table S2: Differentially expressed genes associated with the misfolded protein binding pathway.

**Supporting Information 2**  

## Data Availability

The data that support the findings of this study are openly available in Genome Sequence Archive at https://ngdc.cncb.ac.cn/gsa/index.jsp (Reference Number CRA047551).
