# Supplementary material for: Diosmetin Restores Endoplasmic Reticulum Homeostasis to Ameliorate Podocyte Injury in Diabetic Kidney Disease by Targeting DNAJC3
Source: J Diabetes Res. 2026 Sep 16;2026:5130183. doi: 10.1155/jdr/5130183 (PMC13583059; doi:10.1155/jdr/5130183)
Supplement: Supplementary file 2 [file JDR-2026-5130183-s001.docx]

**Supplementary material**

**1 Reagents**

CMC–Na (C5678-500G) was purchased from Sigma-Aldrich. BCA(A045-4-2) and HE staining assay kit ([D006-1-1](http://www.njjcbio.com/products.asp?id=476)) were purchased from Nanjing Jiancheng Biological Engineering Institute (Nanjing, China). PAS (C0142S) was purchased from Beyotime Biotechnology (Shanghai, China). SOD (A001-3-2), GSH-Px (A005-1-2) and MDA (A003-1-2) were purchased from Nanjing Jiancheng Biological Engineering Institute (Nanjing, China). DNAJC3 (26721-1-AP) and GRP78 (11587-1-AP) were purchased from Proteintech. Podocin (PA5-79757), Phospho-eIF2α (3398S), eIF2α (5324S), ATF4 (11815S) and CHOP (2895S) were purchased from Cell Signaling Technology, Inc..

**2 Supplementary methods**

**2.1 Transcriptomics**

Total RNA was extracted from MPC5 cells using TRIzol reagent (Invitrogen, USA) according to the manufacturer's instructions. RNA integrity was further evaluated using the RNA Nano 6000 Assay Kit on the Bioanalyzer 2100 system (Agilent Technologies, CA, USA). Only samples with an RNA integrity number (RIN) ≥ 8.0 were used for library construction. Subsequently, mRNA was purified from total RNA using poly-T oligo-attached magnetic beads. followed by fragmentation and cDNA synthesis. After adenylation of the 3′ ends of DNA fragments, adaptors were ligated, and fragments of 370–420 bp were selected using AMPure XP beads. Library quality was assessed on the Agilent Bioanalyzer 2100. The clustering of index-coded samples was performed on a cBot Cluster Generation System using TruSeq PE Cluster Kit v3-cBot-HS (Illumina). Sequencing was performed on an Illumina NovaSeq platform. A total of approximately 6 Gb of clean bases (≥ 20 million clean reads) per sample were obtained, with Q30 ≥ 97%. Raw reads in FASTQ format were processed using fastp (v0.20.0) to remove adapter sequences, poly-N-containing reads, and low-quality reads. Clean reads were aligned to the Mus musculus reference genome (mm10/GRCm38) using HISAT2 (v2.0.5). Gene expression levels were quantified using featureCounts (v1.5.0-p3), and FPKM values were calculated. Differential expression analysis between groups was performed using the DESeq2 R package (v1.20.0). Genes with an adjusted P-value < 0.05 and |log₂ fold change| > 1 were defined as differentially expressed genes (DEGs). GO and KEGG enrichment analyses were conducted using clusterProfiler (v3.8.1), with corrected P-value < 0.05 considered statistically significant.

**3 Supplementary tables**

**Table S1. Primer sequence used in RT-qPCR experiment of this study**

| **Gene** | **Species** | **Forward primer (5'->3')** | **Reverse primer (5'->3')** |
| --- | --- | --- | --- |
| *ATF4* | mouse | GTCAACCTATAAAGGCTTGCGG | ACTGCTGCTGGATTTCGTGA |
| *GRP78* | mouse | GGACAAGAAGGAGGATGTGGG | CCGTTCTTGAACACACCGAC |
| *CHOP* | mouse | ACCTGAGGAGAGAGAACCTGG | GGACGCAGGGTCAAGAGTAG |
| *eIF2α* | mouse | CTTCAGAATGCCGGGGCTAA | ACATAGGCCCCCATTTCAGC |
| *Actb* | mouse | TATCGCTGCGCTGGTCG | CCACGATGGAGGGGAATACAG |

**Table S2.** **Differentially expressed genes associated with the misfolded protein binding pathway**

| Gene name | Model *vs.* Vehicle | | DIO *vs*. Model | |
| --- | --- | --- | --- | --- |
|  | log2 (Fold Change) | p-value | log2 (Fold Change) | p-value |
| *AABR07011951.1* | -0.2052 | 0.0002 | 0.3919 | 0.0000 |
| *AABR07033324.1* | -0.1270 | 0.0009 | 0.3108 | 0.0000 |
| *Dnajb9* | -0.7541 | 0.0000 | 1.0290 | 0.0000 |
| *Dnajc3* | -0.5953 | 0.0000 | 1.0971 | 0.0000 |
| *Hspa1b* | -0.4673 | 0.0000 | 1.2349 | 0.0000 |
| *Hspa1l* | -0.5010 | 0.0022 | 0.7585 | 0.0065 |
| *LOC680121* | -0.2596 | 0.0008 | 0.4136 | 0.0000 |
| *Sdf2l1* | -0.1791 | 0.0000 | 0.4928 | 0.0000 |
| *Bag6* | -0.0071 | 0.0788 | 0.0832 | 0.0510 |
| *Edem1* | 0.1267 | 0.0007 | 0.0894 | 0.1303 |
| *Hspa13* | 0.0788 | 0.0955 | 0.0238 | 0.7372 |
| *Clu* | -0.2536 | 0.0000 | -0.0929 | 0.1470 |
| *Dnajc10* | -0.0992 | 0.0033 | 0.0939 | 0.0191 |
| *Hspa14* | -0.2200 | 0.0000 | -0.1087 | 0.0108 |
| *Hspa5* | 0.2197 | 0.0000 | -0.1457 | 0.0000 |
| *Hspa2* | -0.0005 | 0.3558 | -0.0987 | 0.3040 |
| *LOC108348108* | / | 0.4995 | / | 0.4790 |
| *Stub1* | 0.0504 | 0.3487 | -0.1248 | 0.0277 |
| *Tor1a* | 0.0120 | 0.1714 | 0.0527 | 0.3342 |
